# Balancing the critical period of spiking neurons with attractor-less STDP

**DOI:** 10.1186/1471-2202-16-S1-P103

**Published:** 2015-12-18

**Authors:** Simon M Vogt, Ulrich G Hofmann

**Affiliations:** 1 BrainLinks-BrainTools, University of Freiburg, Freiburg im Brisgau, Germany; 2 Neuroelectronic Systems, University Medical Center Freiburg, Freiburg im Brisgau, Germany

## 

Attractor-based ("multiplicative") STDP has been called a biologically more realistic form of spike timing dependent plasticity than attractor-less ("additive") variants [1-4], as it produces unimodal distributions of synaptic weights [5,6] when given poisson-distributed random input spike trains [1,2,4,7-9]. While unimodal weight distributions have been observed as an outcome in some in vitro experiments [5], the actual biochemical process that changes synaptic connection strengths has yet to be fully understood.

Unfortunately, attractor-based STDP has been repeatedly shown to be computationally less powerful than attractor-less STDP [9] and successful implementations of attractor-based STDP used attractors that were either very weak [8] or very close to some minimum weight [2,3], causing a more "additive-like" [2,8] behaviour of the plasticity rule. We therefore examined possible biological interpretations of attractor-less STDP rules while keeping in mind that any STDP rule is just an abstraction from the hidden biophysical reality.

We show how unimodal weight distributions can reliably result from attractor-less STDP when negative synaptic drift is combined with activity-independent synaptic growth. A bimodal distribution is then only formed when non-random (polychromous [10]) poisson-distributed inputs are presented to a neuron [11]. In practice, this produces a plasticity rule that keeps the postsynaptic neuron unselective and responsive to a broad range of inputs while receiving only random spikes, but quickly allows the neuron's receptive field to become highly selective as soon as some inputs start repeating a non-random ordering of spikes.

This stabilization procedure preserves STDP's sensitivity to temporally shifted correlations in input spike data, which in turn gives us several beneficial features for biologically more realistic and computationally more powerful [12] implementations of plasticity in spiking neural networks.
